# Effects of Raised Ambient Temperature on the Local and Systemic Adaptions of Maize

**DOI:** 10.3390/plants11060755

**Published:** 2022-03-11

**Authors:** Zhaoxia Li, Juren Zhang

**Affiliations:** Key Laboratory of Plant Development and Environment Adaptation Biology, Ministry of Education, School of Life Sciences, Shandong University, Qingdao 266237, China; jrzhang@sdu.edu.cn

**Keywords:** maize, heat stress, heat stress response, local and systemic level adaptations

## Abstract

Maize is a staple food, feed, and industrial crop. One of the major stresses on maize production is heat stress, which is usually accompanied by other stresses, such as drought or salinity. In this review, we compared the effects of high temperatures on maize production in China. Heat stress disturbs cellular homeostasis and impedes growth and development in plants. Plants have evolved a variety of responses to minimize the damage related to high temperatures. This review summarized the responses in different cell organelles at elevated temperatures, including transcriptional regulation control in the nuclei, unfolded protein response and endoplasmic reticulum-associated protein quality control in the endoplasmic reticulum (ER), photosynthesis in the chloroplast, and other cell activities. Cells coordinate their activities to mediate the collective stresses of unfavorable environments. Accordingly, we evaluated heat stress at the local and systemic levels in in maize. We discussed the physiological and morphological changes in sensing tissues in response to heat stress in maize and the existing knowledge on systemically acquired acclimation in plants. Finally, we discussed the challenges and prospects of promoting corn thermotolerance by breeding and genetic manipulation.

## 1. Introduction

Maize (*Zea mays* L.), a globally cultivated staple food, feed, and industrial crop, plays a critical role in supporting the nutritional demands of a growing world population. High temperatures are a major stress in maize production and are usually accompanied by other stresses, such as drought or salinity [1]. According to the current trends and predictions of future greenhouse gas emissions, the global average temperature is expected to increase by 1.1–5.4 °C by 2100 (source: Climate.gov, https://www.climate.gov/news-features/understanding-climate/climate-change-global-temperature-projections, (accessed on 18 January 2022)). Record temperatures have been reported at various locations across the globe. The year 2020 was tied with 2016 for the hottest year on record since record-keeping began in 1880, and 19 of the hottest years have occurred since 2000 (source: NASA/GISS, https://climate.nasa.gov/vital-signs/global-temperature/, (accessed on 18 January 2022)). It is estimated that for every 1 °C increase in the mean global temperature, wheat yields will decrease by 6%, rice by 3.2%, maize by 7.4%, and soybean by 3.1% [2]. Plant scientists and breeders now face a time-sensitive challenge in understanding how plants, especially crops, sense and tolerate acute high temperatures.

Although the term heat stress is broadly used, it encompasses heat shock, heat wave, and warming experiments, which vary in the duration and magnitude of the imposed temperature stress. The effects of heat stress may be more pronounced during certain stages of plant growth. In maize production, a hot day above 35 °C during the reproductive stage can cause significant decreases in grain yield. Maize is planted throughout the world. In the northern hemisphere, production is mainly concentrated between the 30° and 50° latitudes, including the United States corn belt and the Northeast and North Plain maize production regions (Huang-Huai-Hai Plain) of China (source: http://www.worldagriculturalproduction.com/crops/corn.aspx, (accessed on 18 January 2022)). Iowa, commonly known as the “corn state,” is considered to have the most suitable soil, moisture, and temperature for corn growth, and is used in this review to compare the relationship between corn production and temperature (high temperature stress). In this review, we compared the effects of high temperature on maize production in China, summarized the developments in maize heat tolerance research, and discussed the opportunities for corn heat-tolerance breeding. The transcriptional regulatory network of plant heat stress response in plants, especially Arabidopsis, was comprehensively reviewed in recent studies [3,4,5] and the heat-stress responses in maize will be discussed in this review.

## 2. Temperature Changes in Maize Production Regions in China

**Figure 1:**
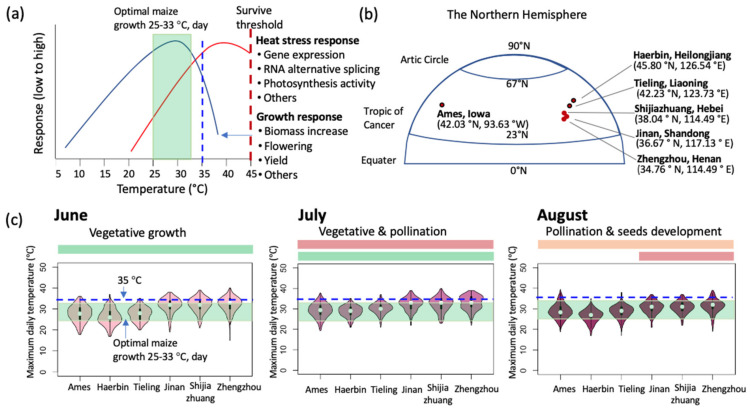
The relationship of temperature, growth, and heat stress in maize and the maximum temperature during maize vegetative growth and reproduction (June, July, and August) in six typical cities in the Chinese maize production region and the US corn belt. (**a**) Optimal growth conditions overlap with the onset of heat stress. The optimal temperature range for maize growth is 25–33 °C day, (green box)/17–23 °C, night. The trends of heat stress and growth responses shown here are from our experiments [7,8] conducted with the maize inbred line W22 grown under controlled environmental conditions in the Enviratron at Iowa State University. (**b**) The latitude distribution of the six cities for the comparison. Ames, United States corn belt; Haerbin and Tieling, in the Northeast maize production region of China, while Jinan, Shijiazhuang, and Zhengzhou in the Huang-Huai-Hai maize production region of China. (**c**) Violin plots show the maximum daily temperature during maize vegetative growth and reproduction (June, July, and August) in six typical cities in the Chinese maize production region and the US corn belt. Violin plots depict distributions of the 10 years’ maximum daily temperature in June, July and August collected for six cities used. Each violin plot is a combination of a box plot and a kernel density plot. In the middle of each density curve is a small box plot, with the rectangle showing the ends of the first and third quartiles and central dot the median. The width of each curve corresponds with the approximate frequency of data points in each region. The light green boxes in panels (**a**,**c**) indicate the range of the optimal maize growth temperature in the daytime. The blue dashed lines represent 35 °C. Historical temperatures for Ames were sourced from https://www.wunderground.com/history (accessed on 18 January 2022) and the historical temperatures for the Chinese cities were sourced from http://tianqi.2345.com (accessed on 18 January 2022).

Maize originated in south-central Mexico and spread throughout the Americas from low-latitude to high-latitude regions [6]. During this acclimation, temperature became one of the most important factors controlling not only arable distribution but also the growth and yield of maize. The optimal temperature range for maize growth is 25–33 °C by day and 17–23 °C at night (Figure 1a) and the optimal temperature for maximum grain yield is approximately 25 °C (source: https://www.extension.purdue.edu/extmedia/nch/nch-40.html (accessed on 18 January 2022)). Interestingly, we found that the onset of heat stress during vegetative growth appears to lie within the range of optimum growth conditions (Figure 1a) based on the analysis of maize plants growth, gene expression, RNA alternative splicing, and flowering time by using a controlled environment facility called the Enviratron to simulate field conditions with different maximal daily temperatures [4,7,8]. The overlap between growth and heat stress suggests that there is a tradeoff between the two, and at temperatures below some optimums, growth dominates, and above that, heat stress outweighs growth. The reproductive phase of maize development is particularly sensitive to heat stress and causes grain yield loss [9,10,11,12,13,14,15]. July and August are the two hottest months in the Northern Hemisphere and often coincide with drought and/or salinity that equates to a reduced overall yield. When extreme heat occurred in the US in 2012 during early grain filling, the overall yield was reduced by 13% compared with 2011 (source: USDA year report, 2013, https://www.nass.usda.gov/Newsroom/archive/2013/01_11_2013.php, (accessed on 18 January 2022)).

**Figure 2:**
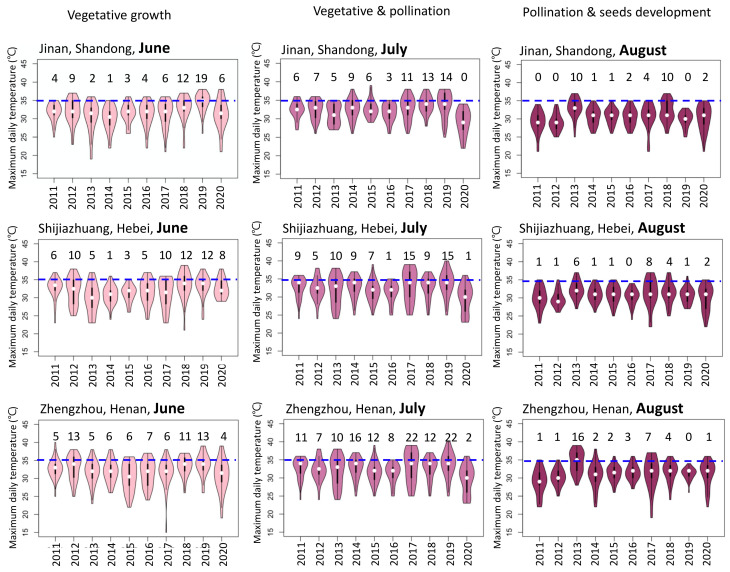
The maximum temperature during maize vegetative growth and reproduction (June, July, and August) in three cities in the Huang-Huai-Hai maize production region of China. The blue lines represent 35 °C, and the numbers represent the numbers of days above 35 °C in that month. Violin plots were generated for the temperatures during 2011–2020. Historical temperatures for the three cities were sourced from http://tianqi.2345.com (accessed on 18 January 2022). See legend in Figure 1c for the explanation of violin plots features.

Compared with the US, the impact of heat stress on maize production in China is much more severe, especially in the Huang-Huai-Hai Plain that has more hot days. As shown in Figure 1b,c, the range of the maximum daily temperature in Ames, in June, July, and August was 15.6–27.8 °C, 17.8–28.9 °C, and 16.7–28.3 °C, respectively, similar to the northeast maize production region in China (Tieling and Haerbin). In Iowa and the northeastern maize production region in China, maize was planted in May, pollinated at the end of July, and filled in August. However, it is quite different in the Huang-Huai-Hai Plain, as the median maximum daily temperature in June and July in Jinan, Zhengzhou, and Shijiazhuang was approximately 33 °C—above the optimum temperature for maize yield. High temperatures in this region are especially detrimental during July when the differentiation of the male and female reproductive systems and pollination occur. Compared with 2011–2015, the number of days in July with temperatures higher than 35 °C increased significantly during 2017–2020 in the maize production region in China (Figure 2 and Figures S1–S3. High temperatures limited overall growth and impaired meiosis in both male and female organs, delay silking, reduced pollen and filament number, impaired pollen germination and pollen tube growth, reduced ovule viability, reduced the number of pollen grains retained by the stigma, disturbed fertilization processes, and impeded the development of endosperm and proembryos, collectively causing grain yield loss [11,12,13,14]. Plant heat tolerance clearly depends on genotypic parameters, especially in maize. Usually, maize inbred lines or hybrids with more tropical germplasm are more tolerant; however, inbred lines used in major maize regions in the Northern Hemisphere are temperate germplasms that are usually sensitive to supra-optimal temperatures. Maize cultivated in different regions could vary greatly in their thermotolerance, which introduces the possibility of selecting and developing climate-resilient maize germplasms.

## 3. Maize Response to Heat Stress at the Cellular Level

The mechanisms of plant heat tolerance have been extensively studied, but few reports exist for maize. Understanding the impact of high temperature stress on maize has important theoretical and practical implications for developing heat-resilient maize germplasm breeding/selection strategies. Similar to other plants, the heat stress response of maize can be summarized at the cellular and tissue level (local level) and plant level (system level). Cells are the basic unit of life and are responsible for all life processes, including the plant stress response. Cells work collaboratively to maintain homeostasis in unfavorable environments. Cell components, including nuclei, chloroplasts, mitochondria, and others, show unique responses when subjected to elevated temperatures.

### 3.1. Heat Stress Responses (HSRs) in the Nuclei/Cytoplasm

**Figure 3:**
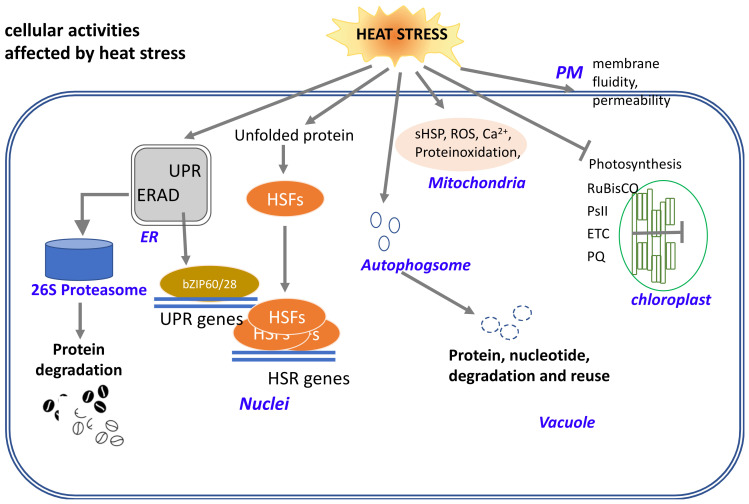
Schematic diagram illustrating the plant response to heat stress at the cellular level. Cell components, including nuclei, chloroplasts, mitochondria, and others, have their own acclimation when subjected to elevated temperatures. HSR in the nuclei/cytoplasm, UPR, and ERQC in the ER, photosynthesis in the chloroplasts, and other cell organelles in response to heat stress are illustrated.

Gene-expression control is a well-defined heat stress response (HSR) that takes place in the nucleus/cytoplasm, especially with regard to genes regulated by heat stress (Figure 3). Heat shock transcription factors (HSFs) and heat shock proteins (HSPs) play pivotal roles in heat stress responses, which mitigate the damage from heat stress and protect plants from further stress. HSF activity is regulated at multiple levels, including at the transcriptional, post-transcriptional, translational, and post-translational levels (reviewed by [4,16,17], and activates the expression of constellations of HSPs. The activation of class A1 HSFs (HSFA1s) is believed to occur as follows: under non-stress conditions, HSFA1s are sequestered in the cytoplasm by their association with HSP90/70 and their cochaperones; in response to heat stress, misfolded proteins recruit chaperones away from HSFs [18], the liberated HSFs undergo trimerization and are imported into the nucleus [19]; and HSF trimerization promotes the ability of HSFs to bind to heat-shock response elements on HSP gene promoters and activate their transcription [20,21]. All higher plants contain moderately sized families of HSFs and are grouped into classes A, B, and C, based on the conserved modular structures [22,23,24,25,26]. Plant HSFs have an N-terminal domain bearing helix-loop-helix DNA-binding motifs and oligomerization domains with bipartite heptad patterns of hydrophobic amino acid residues (HR-A/B region) connected by a flexible linker [22,23,24,27,28]. Class A HSFs have short peptide motifs (AHA motifs) in their C-terminal domains that function as transcriptional activators, while class B and C HSFs do not have AHA motifs or activator functions [26,29]. Most plant HSFs are regulated by heat stress, including up- and down-regulation. For example, Arabidopsis AtHSFA2 [30] and maize Hsftf13 [7] are quickly and rapidly induced by heat. Typically, plant HSFs specifically bind to heat stress elements (HSEs) in the target promoters, and subsequently activate the transcription of stress-inducible genes [26,31]. HSFs activate the expression of heat-stress response genes, including HSPs. This process plays a central role in thermotolerance, transgenerational thermomemory, and many other stress responses in plants. Based on the role of central regulators in heat stress responses, plant HSFs could also be used for gene manipulation.

In addition to the canonical HSR identified in plant heat stress, other factors involved in stress response were also reported in heat stress-induced gene expression reprogramming [3]. In Arabidopsis’ heat-shock response, HsfA1s induce the expression of dehydration-responsive element-binding protein 2A (DREB2A), multiprotein-bridging factor 1c (MBF1c), and other HSFs to upregulate or mediate the expression of heat stress-inducible genes and constitute a complex regulatory network [3]. Histone modification and miRNAs have also emerged as factors involved in transcriptional regulation and heat-stress memory [3]. In a maize transcriptome study of four heat-tolerant and four heat-susceptible inbred lines, 607 heat-responsive genes and 39 heat-tolerant genes were identified [32]. In a transcriptome related to thermotolerance in two sweet corn lines, the Gene Ontology terms for the biosynthesis of secondary metabolites, upregulation of photosynthesis, and downregulation of ribosome function all correlated with improved heat resistance [33]. Nine HSFs were upregulated at elevated temperatures in the leaves of the maize inbred line W22 (at stage V4 and V5, grown in an Enviratron under controlled conditions), including one type A HSF, Hsftf13 (Zm00001d027757), and HSR activation correlated with increasing temperatures [7]. Thirty-one HSFs have been identified in maize based on their sequence similarity, and their expression patterns have been analyzed [24,25]. However, with it being an important crop plant, we still lack a comprehensive understanding of the regulatory mechanisms and natural variation in HSR of the different maize lines.

### 3.2. Unfolded Protein Response (UPR) and Endoplasmic Reticulum-Associated Protein Quality Control (ERQC) in the Response to Heat Stress

The endoplasmic reticulum (ER) is a large, structurally complex organelle that is the main production site for lipids and many proteins and is the entry point for the secretory pathway. Many proteins are introduced into the ER via cotranslational synthesis and folding. Heat induces the UPR and ER-associated degradation (ERAD), which are thought to mitigate the damage caused by heat stress and protect plants against further stress. The UPR upregulates the expression of a canonical set of genes to resolve the protein-folding problem by enhancing ER protein imports, folding, quality control, and exports. The UPR genes in plants are upregulated by the activation of stress-transducing transcription factors [34] via two branches of the UPR signaling pathway, including inositol-requiring enzyme 1 (IRE1) mediated bZIP60 splicing, regulated IRE1-dependent RNA decay (RIDD), and two ER membrane-anchored transcription factors, bZIP17 and bZIP28 [34]. Both bZIP60 and bZIP28 play key roles in the UPR-mediated gene regulatory network under heat-stress conditions [7,35,36,37,38], thereby activating the expression of downstream genes and chaperones, such as binding immunoglobulin protein (BIP), ER-localized DnaJ 3, calnexin/calreticulin, protein disulfide isomerase, and ER oxidoreductase 1 to alleviate the misfolded protein accumulation. We have shown in another study that bZIP60 appears to have been under selection during maize domestication and acclimatization as higher expression levels have been observed in tropical compared to temperate maize lines. These differences appear to be the result of the insertion of transposable elements upstream of bZIP60 [39].

ERAD plays an important role in restoring ER homeostasis when the plant is subjected to common or heat-related environmental stress. Most of our knowledge regarding the mechanisms of ERAD derives from yeast and mammal studies. The ERAD system directly recognizes misfolded proteins and mediates their degradation by proteasomes; both conserved and plant-specific ERAD components have been identified in different ERAD steps [40,41]. Heat stress enhances alternative RNA splicing in plants, including maize [8]. When subjected to heat stress, both abnormal RNA and truncated proteins from the alternative spliced RNA isoforms are accumulated in the cells. This leads to the activation of nonsense-mediated mRNA decay (NMD) and protein degradation, including ERAD, that mitigates toxicity. The ER membrane-anchored E3 ubiquitin ligases CER9 and HRD1A/1B play negative roles in heat-stress responses in Arabidopsis, whereby CER9 and HRD1A/1B altered both the UPR and HSR [42] and increased plant tolerance to heat. Defects in HRD3A of the HRD1/HRD3 complex of the ERAD pathway altered the UPR, increased plant sensitivity to salt, and increased the retention of ERAD substrates in plant cells [43]. Another ERAD component, ubiquitin-conjugating enzyme (UBC) 32, functions as a positive regulator of drought tolerance and the ABA response by modifying the stability of two aquaporin proteins, namely PIP2;1 and PIP2;2 [44]. Although evidence is lacking in maize, these ERAD components may play major roles in mediating the ubiquitination of misfolded proteins and are potentially involved in abiotic stress responses, including heat stress.

### 3.3. Photosynthesis in the Chloroplasts

Biomass production of higher plants largely depends on leaf photosynthesis that occurs in the chloroplasts. Chloroplasts serve as metabolic centers and play a key role in physiological adaptations to abiotic stresses. Photosynthesis rates vary with temperature. Generally, the thermal optimum is approximately equal to the average temperature of a suitable environment. It has long been recognized that C4 plant species have a higher optimum temperature for photosynthesis than C3 plants [45,46]. In a previous report, net photosynthesis in maize exhibited a broad range of optimal temperatures between 28 °C and 37.5 °C covered the optimal temperature range for maize growth (25–33 °C, day). The activation of Rubisco decreased at temperatures exceeding 32.5 °C, with a nearly complete inactivation at 45 °C, and the relative inhibition was much greater when the leaf temperature increased rapidly compared with when it was gradually increased [47]. Heat stress reduces the photosynthetic capacity and carbon assimilation rate of maze leaves [48,49] due to the sensitivity of the photosynthetic apparatus to heat, reduced photosynthetic electron transfer, Rubisco activation, and accelerated chlorophyll breakdown and other structural changes in the chloroplast [50,51,52,53]. In maize, heat stress can cause swelling of the chloroplasts with reduced granule stacking [51,53,54], aggravating oxidative damage [50,51,53] and inducing the photoinhibition of photosystem II (PSII) [55]. A decline in the chlorophyll level has been observed under heat stress due to an increase in chlorophyllase and Chl-degrading peroxidase activity [56]. This is the primary constraint on the rate of photosynthesis when the maize leaf temperature is above 30 °C. The photosynthetic apparatus could be acclimated to heat stress by, for example, improving antioxidant capacity and metabolism [57]. However, a case study using four maize commercial elite hybrids showed a sharp decline in the photosynthetic rate of 10% per 1 °C, at air temperatures greater than 40 °C; at 45 °C, some genotypes of maize retained ~40% of the maximum relative photosynthetic rate [52].

PSII is a particularly heat-sensitive component of the photosynthetic apparatus [46,58]. Heat stress causes photoinhibition of PSII, but not PSI. Heat stress damages the oxygen-evolving complex, disturbs electron transfer from plastoquinone QA to QB, and inhibits the repair of PSII via the degradation of the QB-binding protein [53,59,60]. The attack of reactive oxygen species (ROS) during moderate heat stress principally affects the repair system of PSII, but not directly the PSII reaction center, which would only be damaged at high temperatures, most commonly at temperatures above 45 °C [61,62,63,64,65]. In maize, the increase in temperature caused the gradual inhibition of whole chain electron transport and at 40 °C of incubation 52% loss was noticed [66]. The 40 °C treatment caused a 55% loss in electron transport activity of thylakoid membranes [66].

### 3.4. The Response of Other Cellular Components to Heat Stress

Electrolyte leakage is a method used to detect the level of injury in plants under abiotic stress and is used to evaluate heat tolerance in different cultivars and species. Heat stress dramatically increases the cellular membrane permeability and leads to a loss in electrolytes, consequently inhibiting cellular function and decreasing cell vitality [67]. The ROS accumulation caused by heat stress leads to membrane damage and decreases the thermotolerance of plants [68]. Similar to mammalian cells, heat stress also induces the production of ROS by the mitochondria, resulting in intracellular oxidative stress, which is a complex process that induces mitochondrial DNA mutations and lipid peroxidation, leading to a loss of mitochondrial membrane potential and overall function [69]. Mitochondrial swelling and ultrastructural disorganization occur in plants under heat stress. Changes in ambient temperature are perceived by cells with a complicated set of sensors located in various cellular compartments. However, it remains unclear how the cell coordinates interactions and communicates between the multiple cellular compartments, including the plasma membrane, cytosol, energy-associated organelles, and chromatin, which maintain homeostasis during adverse conditions, especially in crop plants. There is widespread support for the notion that an increase in plasma membrane fluidity, induced by high temperatures activates stress-signaling pathways via different integral membrane proteins (i.e., channels, transporters, and receptor-like kinases) and various secondary messengers, such as Ca^2+^ ions, nitric oxide, hydrogen peroxide, and phytohormones, to trigger the expression of certain genes and increase the accumulation of beneficial metabolites to enhance heat tolerance [70].

Autophagosomes are temporary organelles that only exist in cells when autophagy occurs. Selective autophagy is known to function in protein quality control by targeting the degradation of misfolded and potentially toxic proteins. Although its role and regulation in the heat-stress responses of crop plants are not clear, autophagy is induced by abnormally high maximum daily temperatures and follows the same diurnal temperature patterns as other UPR indicators [7]. The compromised heat tolerance of autophagy (*atg*)*5*, *atg7*, and a neighbor of breast cancer (*nbr1*) mutants has also been associated with an elevated accumulation of insoluble, detergent-resistant proteins that were highly ubiquitinated under heat stress [71]. Autophagy assists in resetting the cellular memory of heat stress in Arabidopsis and mediates the specific degradation of heat shock proteins at later stages of the thermorecovery phase, leading to the accumulation of protein aggregates after the second phase of heat stress and a compromised heat tolerance [72,73].

## 4. Maize Response to Heat Stress—Local to Systemic

Being sessile organisms, only a part of the plant body has to sense the changing environmental conditions before the rest of the plant responds, such as the wilting of leaves in high ambient temperatures and the growth of a root tip at the season’s first rain. Usually, the sensing tissue (local) generates a signal that is broadcasted through the plant body (systemic) and triggers acclimation or defense-related processes to manage the adverse environmental conditions. Systemic responses to environmental stimuli are essential for the survival of multicellular organisms, especially outdoor crops. As a monoecious organism, maize develops unisexual male and female flowers in separate parts of the plant. Heat stress affects the synthesis of carbohydrates in the leaves and disrupts the anthesis-silking synchrony, reduces pollen grain viability, inhibits ovule fertilization, and increases kernel abortion, resulting in severe yield losses [9,10,15,74]. High-temperature damage to maize typically coincides with drought stress and, in combination, presents a more severe challenge to maize growth than a single stress. However, both stresses induce metabolite accumulation and trigger the production of enzymatic and non-enzymatic antioxidants to prevent oxidative damage. The successful selection and breeding of heat-tolerant maize cultivars require a comprehensive understanding of both local and systemic responses to elevated temperatures.

**Figure 4:**
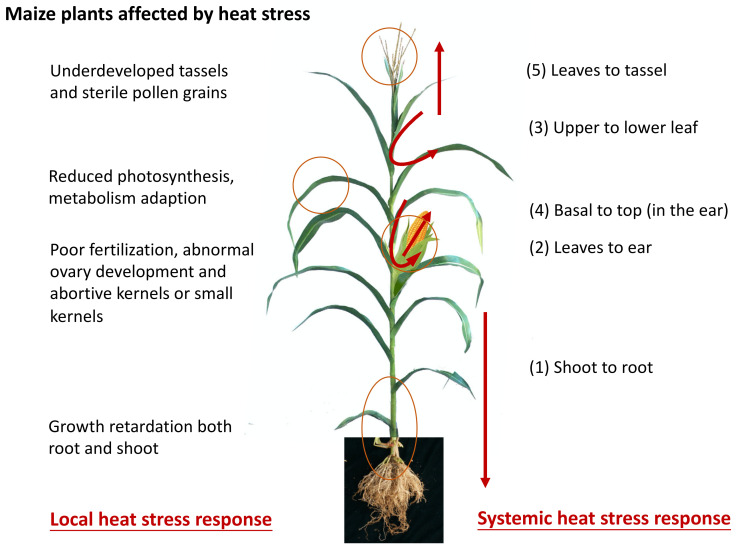
Schematic diagram illustrating the local and systemic level responses to heat stress.

The sensing tissues that respond to heat stress (local level) have previously been elucidated by physiological and morphological analyses (Figure 4). In maize, the period of reproductive development is the stage in which it is most vulnerable to heat stress and coincides with peak temperatures in the growing season. Heat stress reduces the photosynthetic capacity of maize leaves [7,47,50,51,52,54], which in turn reduces the source capacity. The location of the tassel, on top of the plant, implies a high degree of exposure to heat and scorching winds. During tassel initiation to tassel emergence, high temperatures cause a delay in silking more than anthesis [75], damage tassels, desiccate pollen, and can lead to silk death [76], which results in de-synchronization and reduced fertilization. When subjected to excessive heat, tassel sterility or tassel blasting (drying of part or the complete tassel without pollen extrusion) will occur and dramatically reduce the yield. For the ear, heat-induced unsuccessful fertilization and extreme temperatures affect grain development and reduce yields. Although heat stress on maize production has attracted a lot of attention from both plant scientists and breeders in recent years, systemic responses, which are involved in producing signals to elicit responses in remote plant parts, remain unclear. For example, it is unclear how plants detect the ambient temperature to coordinate multiple organs, including source leaves, tassel, ear, and root systems to cope with the ambient temperature.

In plants, systemically acquired acclimation (SAA) is thought to be controlled by a rapid systemic signaling mechanism that maintains homeostasis during adverse environmental conditions. The rapid and systemic whole-plant transmission of electric, calcium, and ROS waves is required for plant acclimation to heat, light stress, and wound responses [77,78,79,80,81]. ROS play a fundamental dual role in plants; they are toxic to cells, causing oxidative stress, but are also important signaling molecules during a stress response. Heat-stress induced bursts of ROS generated by a local group of plant cells (such as the local leaves subjected to heat) results in the formation of a wave of ROS production that propagates throughout the different tissues of the plant and carries a systemic signal over long distances; the ROS wave was found to function in coordination with ABA in systemic tissues [78]. The accumulation of jasmonic acid and salicylic acid in local leaves plays an important role in systemic signal integration during the presence of combined environmental stresses [82]. Active SAA participates in both basal and acquired thermotolerance and stress memory. In a high light and heat stress study using Arabidopsis, Zandalinas et al. [82] found that plants can integrate two different systemic signals that were simultaneously generated during combined stress responses. This integrated response significantly influenced the efficiency of systemic ROS signal induction as well as transcriptomic, hormonal, and stomatal responses, and acclimation. In addition, ROS-producing systems are required for the activation of HSF-dependent pathways. The mammalian and Drosophila HSFs directly sense both heat and hydrogen peroxide in order to assemble a homotrimer in a reversible and redox-regulated manner, establishing a mechanism for stress, i.e., active HSP gene expression [83,84]. Compared to other HSFs, plant AtHsfA4a/HSF21 and AtHsfA8 show a consistently higher level of expression in the *apx1* mutant [85] and potentially function as ROS or redox sensors [85,86]. Overall, the SAA and its roles in maize heat tolerance remains unclear and requires further investigation. The mechanisms of signal transport from leaves to tassel, upper to lower leaf, leaf to ear, and shoot to root still need to be elucidated (Figure 4). At present, we do not know which signals function in this process, and the roles of phytohormones, phloem/xylem transport, ROS, and Ca^2+^ in the sensing and transmission of thermal stress signals are important topics for exploration in future research.

## 5. Conclusions, Prospects, and Maize Breeding

Maize is an important crop worldwide. Its yield is significantly affected by drought and heat stress and plants are bound to deal with heat or temperature stress due to the expected increase in average global temperatures. Improving heat tolerance in maize has become one of the top priorities for maize-breeding programs. In maize, a large variation in heat tolerance exists in germplasm collections due to its successful acclimation from the tropics to temperate regions in the Northern and Southern Hemispheres [87,88,89,90,91,92]. The development of heat-tolerant maize hybrids is urgently needed, and this goal largely depends on the study of physiological, biochemical, and molecular mechanisms.

Genomic regions conferring stress tolerance and their association with linked morphological markers can be used to develop stress-resilient lines/varieties via marker-assisted selection (MAS). During the past decades, conventional and modern breeding platforms, including MAS, quantitative trait loci mapping (QTL), and genome-wide association studies (GWAS), have made tremendous progress in the development of heat-tolerant maize hybrids. Selection is based on morpho-physiological changes, grain yield, osmolyte accumulation, nutrient uptake, and oxidative status in maize lines grown under different conditions with heat stress or heat combined with drought stress [93]. Through QTL mapping, six QTLs were identified, each explaining between 7% and 9% of the phenotypic variance [94]. Multiple QTLs conferring heat tolerance, especially at the reproductive stage in maize, have been mapped along with their associated markers using dent and flint maize germplasms [95]. Genetic mapping of foliar and tassel heat tolerance in maize in two biparental recombinant inbred line populations (B73 × NC350 and B73 × CML103) showed that heat tolerance traits are mediated by complex genetic control [96]. Heat tolerance of maize seems to be regulated by a polygenic system, making it genetically complicated to develop heat tolerant genotypes. A GWAS can be a powerful tool in breeding programs because of its ability to efficiently analyze complex traits under different environmental conditions. Six significant haplotype associations for grain yield under combined drought and heat stress management on chromosome 4 were identified by GWAS [97]. In another study, 12 significant single nucleotide polymorphism (SNP) associations were found for grain yield under heat stress from a panel of 662 double haploid lines of tropical origin [98], and they explained about 18% of the phenotypic variation. In a recent study, by using 543 tropical maize inbred lines from diverse genetic backgrounds in nine locations across South Asia under natural heat stress, 175 SNPs were found in 140 unique gene models that were implicated in various biological pathway responses to different abiotic stresses [99]. Phenotyping protocols are essential for evaluating large populations in terms of both selection processes and source identification of tolerance. An understanding of the heat-stress response in maize is needed to help predict and identify the heat tolerance of the lines and to develop markers that can be used in molecular-assisted breeding to increase efficiency.

At the same time, gene modification by overexpression or gene editing can be used directly to change some key genes. There is much more to learn in laboratory manipulation studies and much more that genetic research can do for maize breeding. In summary, heat tolerance is a complex phenomenon that involves several individual events as well as events in conjugation. Among the physiological adaptations, maintaining the optimum photosynthetic rate under high temperature stress is the key physiological process that contributes to heat tolerance. A higher rate of photosynthesis is directly correlated with heat tolerance and, hence, economic yield. The antioxidative stress metabolism in cells comprising enzymatic and non-enzymatic antioxidants imparts stress tolerance by scavenging or detoxification of excess ROS. A better understanding of all the intricacies of the antioxidative stress metabolism will help in designing appropriate strategies to develop crop plants with improved high-temperature stress tolerance. Transcription factors are promising targets for the modification of thermotolerance in maize, as they may function in regulating quantitative traits. The transgenic manipulation of such transcription factors should help us to understand more about multigene regulation and its relationship to tolerance and could be used to develop inbred lines with advanced capabilities for thriving under higher environmental temperatures.

## Data Availability

Not applicable.

## Supplementary Materials

The following supporting information can be downloaded at: https://www.mdpi.com/article/10.3390/plants11060755/s1, Figure S1: The maximum temperature during maize vegetative growth and reproduction (June, July, and August) in Ames, Haerbin, and Tieling. Figure S2: Violin plots shows the maximum daily temperature during maize vegetative growth and reproduction (June, July, and August) in six typical cities in the Chinese maize production region and the US corn belt. Figure S3: The maximum temperature during maize vegetative growth and reproduction (June, July, and August) in Jinan, Shijiazhuang, Zhengzhou, Ames, Haerbin, and Tieling.
