# Supplementary material for: Effects of Raised Ambient Temperature on the Local and Systemic Adaptions of Maize
Source: Plants (Basel). 2022 Mar 11;11(6):755. doi: 10.3390/plants11060755 (PMC8949135; doi:10.3390/plants11060755)
Supplement: Supplementary file 1 [file plants-11-00755-s001.zip › plants-1623665-supplementary.pdf]

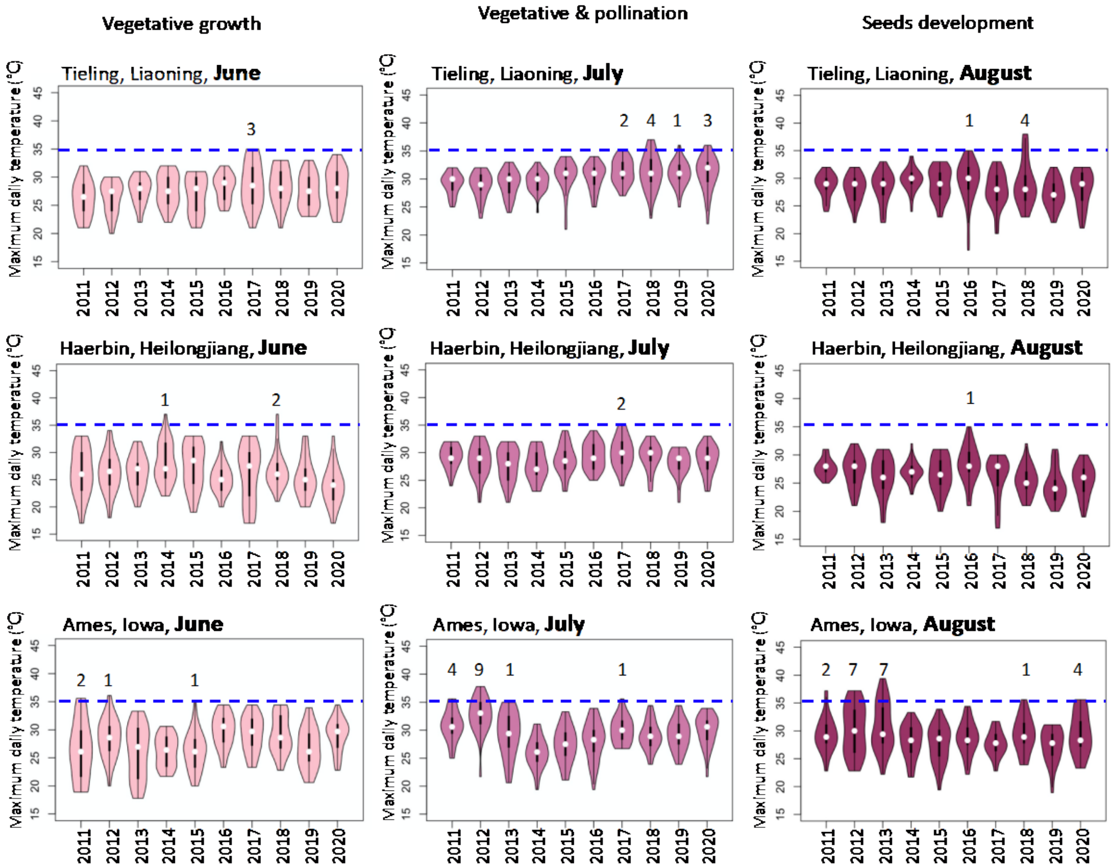

Figure S1. The maximum temperature during maize vegetative growth and reproduction (June, July, and August) in Ames, Haerbin, and Tieling. The blue lines represent 35 °C, and the numbers were the days above 35 °C in that month. Violin plots were generated for the temperatures during 2011–2020. Historical temperatures for Ames were sourced from https://www.wunderground.com/history (retrieved January, 2022) and historical temperatures for the Chinese cities were sourced from http://tianqi.2345.com (retrieved January, 2022). See legend in Figure 1c for the explanation of violin plots features.

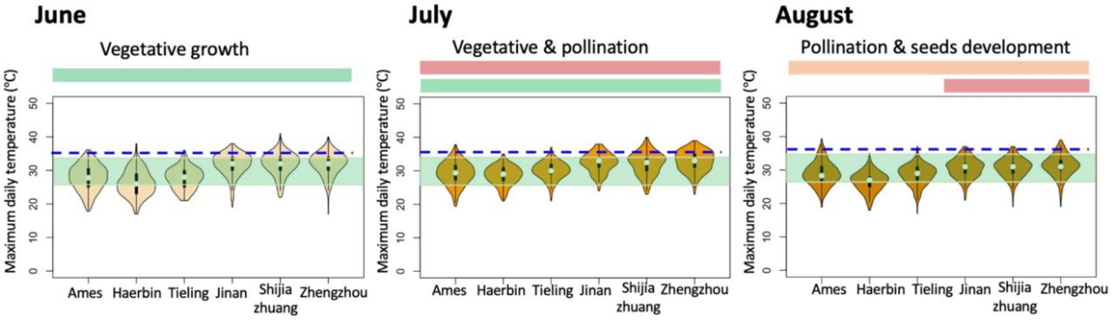

Figure S2. Violin plots shows the maximum daily temperature during maize vegetative growth and reproduction (June, July, and August) in six typical cities in the Chinese maize production region and the US corn belt. Violin plots depicts distributions of the 10 years' maximum daily temperature in June, July and August collected for six cities used. The blue lines represent 35 °C. Violin plots were generated for the temperatures during 2011–2020. The light green boxes in panels indicate the range of the optimal maize growth temperature in the daytime. Historical temperatures for Ames were sourced from https://www.wunderground.com/history (retrieved January, 2022) and historical temperatures for the Chinese cities were sourced from http://lishi.tianqi.com/, (retrieved January, 2022). See legend in Figure 1c for the explanation of violin plots features.

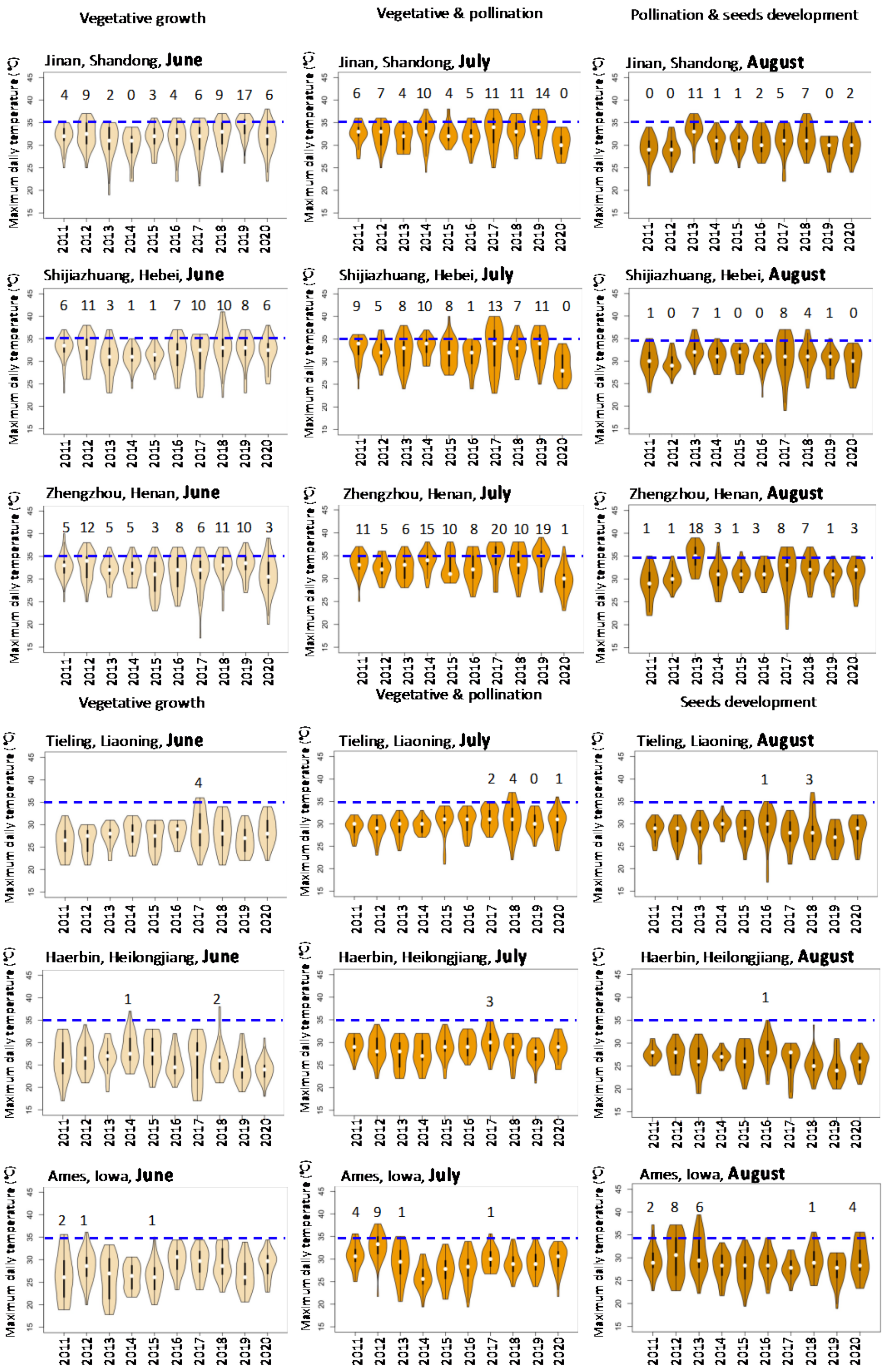

Figure S3. The maximum temperature during maize vegetative growth and reproduction (June, July, and August) in Jinan, Shijiazhuang, Zhengzhou, Ames, Haerbin, and Tieling. The blue lines represent 35 °C, and the numbers were the days above 35 °C in that month. Violin plots were generated for the temperatures during 2011–2020. Historical temperatures for Ames were sourced from

https://www.wunderground.com/history (retrieved January, 2022) and historical temperatures for the Chinese cities were sourced from http://lishi.tianqi.com/, retrieved January, 2022. See legend in Figure 1c for the explanation of violin plots features.
